# Molecular Phylogenetics 2014

**DOI:** 10.1155/2015/919251

**Published:** 2015-06-02

**Authors:** Vassily Lyubetsky, William H. Piel, Peter F. Stadler

**Affiliations:** ^1^Institute for Information Transmission Problems (Kharkevich Institute), Russian Academy of Sciences, 127994 Moscow, Russia; ^2^Yale-NUS College & National University of Singapore, Singapore 138614; ^3^Institut für Informatik, University of Leipzig, 04109 Leipzig, Germany

Knowledge of phylogeny is of fundamental importance for understanding evolution. It has become an indispensable tool in modern genomics as a framework for interpreting genomes and metagenomes, for understanding evolution of genes, proteins, and noncoding RNAs as well as different types of regulations including secondary RNA and protein structures, and for reconstructing ancestral genomes [1]. The era of next-generation sequencing (NGS) brought an influx of data but posed theoretical challenges, for example, in reducing systematic errors and increasing robustness under much less sampling error [2].

An important avenue of modern phylogenetics is the study of coevolution. Here a major focus is the developing of effective algorithms to infer common history of genes, proteins, molecular machines (ribosomes, RNA polymerases, and transporter systems), signal transductions, metabolic pathways, chromosomal structures and gene synteny, and so forth. An important direction is to define and reconstruct the evolutionary scenario on the basis of polytomous trees, also in application to study fast evolving bacteria and viruses with high medical impact, and in shaping the primary genomes. Dating of the phylogeny immediately depends on progress in detecting zones of active horizontal gene transfers or other genomic events, defining time slices to describe tree-like phylogenies, and so forth.

Mathematics of phylogenetics will grow to foster alternative ways to describe evolution: generalization of trees into nets and developing models based on stochastic and partial differential equations. An important focus is to develop low polynomial complexity algorithms for exact models that are usually solved heuristically. Contemporary methods of clustering and discrete optimization have already proved very effective. Thus, graphs with 10^20^ vertices, 10^9^ parts, and the same average vertex degree can be processed on a multiprocessor machine in reasonable time. A high priority for testing modern algorithms is to obtain primary data with known predefined solutions. The call is for realistic computational models that allow simulating large-scale phylogenies and can serve for future studies, as well as real data with known phylogenies or at least few data sets where everybody would agree what the correct phylogeny is.

The contents of the special issue of 2015 cover research of various groups that use the toolkit of phylogenetics to tackle a spectrum of evolutionary questions. Apart from classic molecular systematic applications to infer taxon phylogenies, the trend is obvious to approach molecular and biodiversity assessment at different levels in various communities, at intraspecific level and in environmental samples, including systematic studies of bacterial and viral pathogenic agents. Molecular markers such as mobile elements are being developed and exploited in studies of population polymorphisms, and RNA secondary structures are used to detect signatures of selection.

A historical profile of molecular phylogenetics with some extrapolations into the future, as well as a brief outline of hot spots in this field, can be found in this special issue [3]. We truly hope that these contributions will be of use to scientists in various areas in possibly helping them to find answers and pose new questions in their own research.

*Vassily Lyubetsky* *William H. Piel* *Peter F. Stadler*
